# A colorful legacy of hybridization in wood-warblers includes frequent sharing of carotenoid genes among species and genera

**DOI:** 10.1371/journal.pbio.3003501

**Published:** 2025-12-11

**Authors:** Kevin F. P. Bennett, Andrew W. Wood, Marcella D. Baiz, Lan-Nhi Phung, Irby J. Lovette, David P. L. Toews

**Affiliations:** 1 Department of Biology, Pennsylvania State University, State College, Pennsylvania, United States of America; 2 National Museum of Natural History, Smithsonian Institution, Washington, DC, United States of America; 3 Natural Resources Research Institute, University of Minnesota Duluth, Duluth, Minnesota, United States of America; 4 Department of Biological Sciences, University at Buffalo, SUNY, Buffalo, New York, United States of America; 5 Department of Biology, University of Rochester, Rochester, New York, United States of America; 6 Fuller Evolutionary Biology Program, Cornell Lab of Ornithology, Cornell University, Ithaca, New York, United States of America; University of Cambridge, UNITED KINGDOM OF GREAT BRITAIN AND NORTHERN IRELAND

## Abstract

Introgression between species has the potential to shape evolutionary trajectories in important ways, but uncovering complex introgression dynamics has only recently been made possible by advances in genomics. Warblers of the avian family Parulidae exemplify rapid diversification and sexual trait divergence, and we endeavored to study historical introgression in the family. We sequenced multiple genomes of nearly every species, constructed a phylogeny for the family, and investigated gene flow across the genome and at genes known for controlling feather color. We found that DNA haplotypes including the gene *BCO2*, which encodes an enzyme that degrades yellow carotenoids, have spread among genera multiple times—from *Vermivora* to *Geothlypis* and from *Leiothlypis* to multiple *Cardellina* and *Setophaga* species. Patterns of inheritance in the latter case point to introgression that occurred 0.5 to 2 million years ago, and the shared haplotype among recipient species is less than 100 nucleotides long. Separately, we found evidence of introgression from red *Cardellina* species to both of the two red *Myioborus* species at *BDH1L* and from one red *Myioborus* species to the other at *CYP2J19*; both are key genes in the pathway that converts yellow carotenoids to red ketocarotenoids. Our results show that introgression is a common mechanism for the evolution of colorful plumage in parulid warblers and hint at complex histories of gene flow behind some of the Western Hemisphere’s most colorful birds.

## Abstract 2

Introgression between species can shape evolutionary trajectories in important ways. This study of the entire family of the colorful wood-warblers (Parulidae) uses whole-genome sequencing to reveal frequent intergeneric and interspecific introgression of genes related to carotenoid-based plumage color.

## Introduction

Hybridization—interbreeding between species or divergent populations—has the potential to impact evolution in important ways. Particularly in the genomics era, hybridization has been recognized for its role in introducing adaptive traits to new species through introgression. What were once famous systems illustrating adaptation to new environments, like African cichlids [1], or predator avoidance, like *Heliconius* butterflies [2], have also now taken on new meaning as powerful examples of hybridization’s great potential to shape evolutionary trajectories through introgression [3–6]. Beyond such classic systems in evolution, similar discoveries are emerging from non-model organisms. Adaptive traits now linked to historical introgression include loss of seasonal-white coat color in hares living in areas with warm winters [7] and neurotoxic venom in a rattlesnake species with predominantly hemorrhagic venom [8].

The wood-warblers of North, Central, and South America (family Parulidae) are a promising study system to characterize the evolutionary consequences of historical hybridization and introgression. The parulid radiation of >100 species occurred extremely rapidly, including the largest diversification rate increase relative to the background rate in songbird evolution [9]. Warblers are notable for striking differences in their secondary sexual traits, including male breeding plumage and song, and the apparent ecological similarity of many species that occur in sympatry without common hybridization [10]. Nevertheless, young species form hybrid zones upon secondary contact [11–15], and the group has been noted for producing frequent intergeneric hybrids [16,17], some which have recently been confirmed with genomic data [18,19]. By contrast, historical hybridization has been largely unexplored in warblers, but it may be an important contributor to the family’s evolution. For example, recent research found that the gene beta-carotene 9′,10′-oxygenase 2 (*BCO2*), which codes for an enzyme that cleaves yellow carotenoids [20,21] and underlies color polymorphisms in diverse animal species [22–26], has spread between colorful species multiple times in the genus *Setophaga* [27]. Those preliminary phylogenetic patterns also implied that the introgressed *BCO2* haplotype may have originated outside the *Setophaga* genus [27], raising the intriguing possibility that historical hybridization and introgression may have played a key role in the evolution of plumage color across multiple sub-groups of these warblers. Closer inspection of other coloration genes may reveal that *BCO2* is just one of several with histories of past gene flow. Beyond *BCO2*, the two enzymes with the best characterized roles in avian carotenoid coloration are CYP2J19, a cytochrome P450 oxygenase, and BDH1L, a short-chain dehydrogenase. Both catalyze essential reactions in the conversion pathway from yellow dietary carotenoids to red keto-carotenoids [28,29], and variation in both has been linked to phenotypic diversity of bird colors [28,30,31].

In this study, we used whole-genome sequencing to test for historical gene flow across the entire wood warbler family. We first generated a low-coverage genome resequencing dataset comprised of multiple individuals of nearly every warbler species (*n* = 480 individuals from *n* = 103 taxa), including 17 of the 18 recognized genera in the family. Next, we extracted ultra-conserved elements (UCEs) from the sequencing data and used them to construct a species-level phylogeny of Parulidae. We then examined patterns of gene tree-species tree discordance at the three candidate genes described above. We found clear evidence for repeated past episodes of intergeneric and interspecific gene flow at all three loci. We validated these findings with population genetic statistics and characterized genotype patterns across the regions of interest to identify precise introgression haplotypes. We explored variants in the *BCO2* introgression haplotype and identified potential functional SNPs unique to a set of gene flow recipient species. Finally, we evaluated whether the color gene introgression we discovered reflected genome-wide patterns of gene flow.

## Results and discussion

### Phylogenomics confirms most of the existing parulid taxonomy

**Fig 1:**
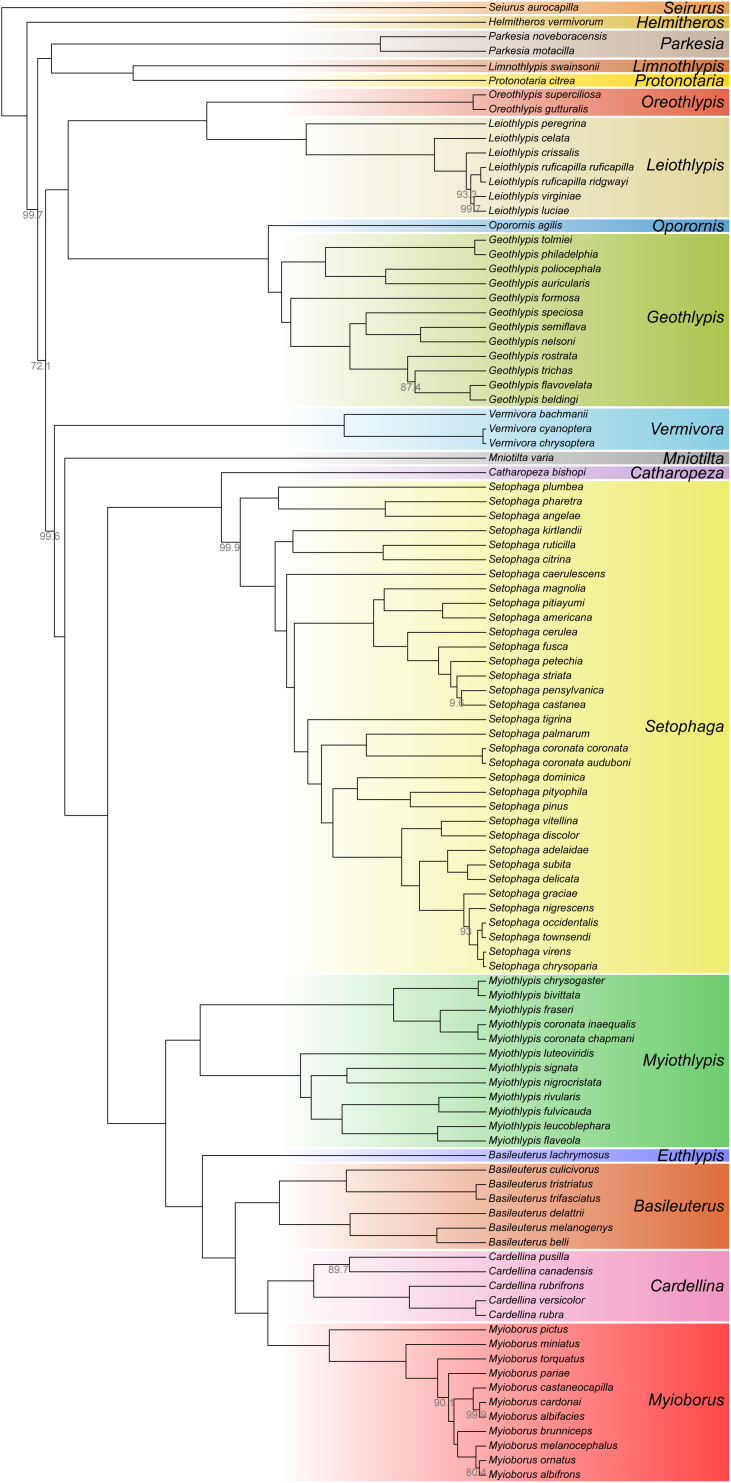
Phylogeny of Parulidae estimated from 4,016 concatenated ultra-conserved element loci. Nodes receiving less than 100% support by approximate likelihood ratio test (aLRT) are labeled with their aLRT value. Tip labels are current species designations based on the eBird/Clements checklist [32]. Genus labels reflect an updated taxonomy implicated by our results and another recent phylogenetic study of the warblers [34]. The data and code needed to generate this figure can be found at https://doi.org/10.5061/dryad.1zcrjdg3v and from NCBI at BioProject PRJNA630247.

We sequenced DNA from blood or frozen tissue of 480 individuals (S1 Table) on Illumina HighSeq and NextSeq platforms across 14 total sequencing runs. Average sequencing coverage was 3.79× per individual. To make the parulid phylogeny, we combined reads from separate individuals into species alignments mapped to the Yellow-rumped Warbler (*Setophaga coronata*) reference (NCBI Accession mywa_2.1), then bioinformatically extracted ~4,000 UCE loci and used them to generate a well-supported phylogenetic hypothesis for Parulidae (Fig 1). The resulting tree contains 100 species and six subspecies of the 115 species in the family, according to the Clements taxonomy [32]. We found strong support for placing *Basileuterus lachrymosus* in its own genus, which aligns with its previous classification, *Euthlypis lachrymosa* [33], but otherwise found support for all existing warbler species classifications. The two tree-building methods we used, concatenation and quartet-based gene tree reconciliation, agreed on most of the phylogeny. There were several differences, however, including relationships among some of the youngest species, as well as whether to place *Catharopeza bishopi* in a monotypic genus sister to the genus *Setophaga* or in a sub-clade of *Setophaga* with several other island endemic warblers (S1 Fig). Another recent warbler phylogeny used UCE target-capture data and a mix of frozen tissue and low-quality toepad samples [34]. Our concatenated tree agreed with it on the placement of every genus, including *Euthlypis*, with the only exception being the placement of the clade containing *Protonotaria* and *Limnothlypis*. We used our concatenated tree in downstream analyses as the warbler species tree (Fig 1).

### Carotenoid metabolism gene *BCO2* shows evidence of repeated intergeneric introgression

**Fig 2:**
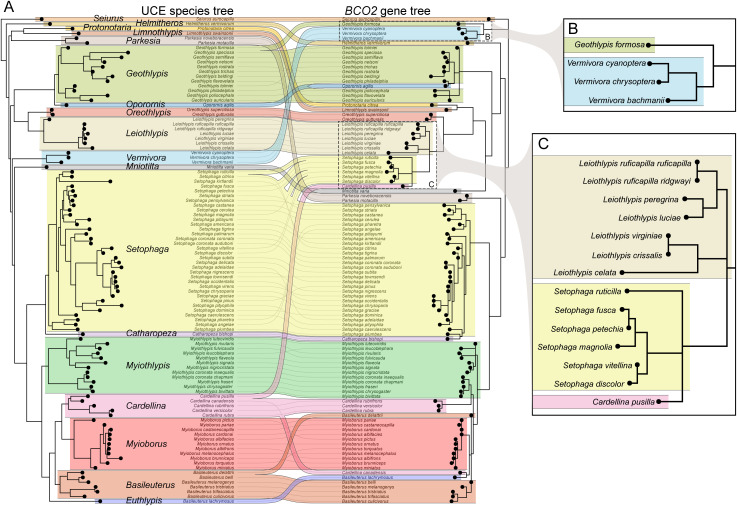
Topological discordance between the concatenated ultra-conserved element species tree of parulid warblers and a 1.8-kb region of the *BCO2* gene. **(A)** Full co-phylogeny, with the species tree on the left and gene tree on the right. **(B)** The *Vermivora* branch of the tree, showing evidence of gene flow to *Geothlypis formosa*. **(C)** The *Leiothlypis* branch of the tree, showing evidence of gene flow to several *Setophaga* and one *Cardellina* species. See S2 Fig for *BCO2* gene tree node support values. The data and code needed to generate this figure can be found at https://doi.org/10.5061/dryad.1zcrjdg3v and from NCBI at BioProject PRJNA630247.

We previously showed that the gene for carotenoid metabolism enzyme *BCO2* has introgressed among species in the genus *Setophaga* several times and that the introgressed variant may have originated outside the genus [27]. We were therefore motivated to study gene flow at *BCO2* across the entire family to understand the full history of *BCO2* evolution in warblers. First, we examined gene tree-species tree discordance at the *BCO2* locus. The tree derived from a 1.8-kb region within the gene that contained 862 SNPs showed considerable discordance with the species tree, including multiple instances of discordance among genera (Figs 2A and S2). The most striking discordances from this analysis were (1) *Geothlypis formosa* falling on the *Vermivora* branch of the gene tree and (2) *Cardellina pusilla* falling on the *Leiothlypis* branch of the gene tree, along with six species of *Setophaga* (Fig 2B and 2C). This topology is consistent with (1) historical gene flow from *Vermivora* to *Geothlypis* and (2) from *Leiothlypis* to *Setophaga* and *Cardellina*, or from *Leiothlypis* to *Setophaga* and then from *Setophaga* to *Cardellina* or vice versa.

**Fig 3:**
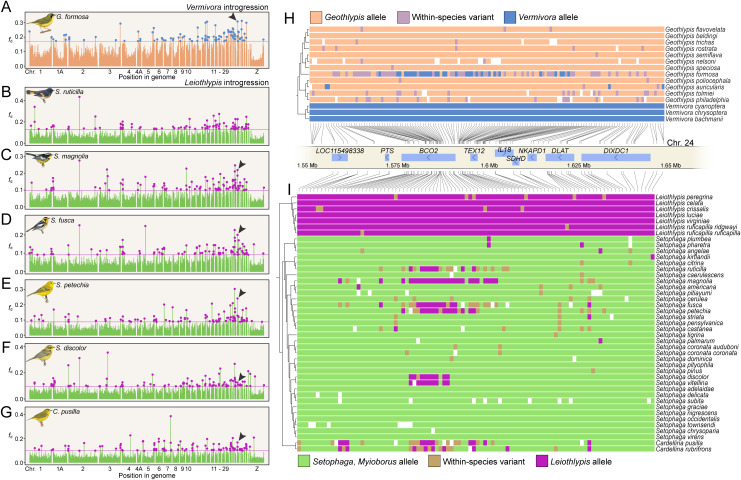
*BCO2* introgression in parulid warblers. **(A–G)** Plots of introgression metric *f*_d_ in 10-kb windows with *Vermivora cyanoptera* (A) or *Leiothlypis ruficapilla* (B–G) as the presumed “donor” species and the recipient labeled on each plot. Black arrows indicate where a window within or adjacent to *BCO2* is a 99.9% outlier. **(H and** **I)** Plots showing the genotype at biallelic SNPs in the *BCO2* region. (H) Sites with ≥90% frequency of one allele in *Vermivora*, ≥90% frequency of a different allele in *Geothlypis* (omitting *G. formosa*), and <10% missing data. (I) Sites with ≥90% frequency of one allele in *Leiothlypis* and ≥90% frequency of a different allele in *Setophaga* and *Myioborus* (omitting *S. ruticilla*, *S. magnolia*, *S. fusca*, *S. petechia*, *S. discolor*, and *S. vitellina*). Warbler illustrations courtesy of Cornell Lab of Ornithology, illustrated by David Quinn, from Birds of the World (https://doi.org/10.2173/bow). The data and code needed to generate this figure can be found at https://doi.org/10.5061/dryad.1zcrjdg3v and from NCBI at BioProject PRJNA630247.

To support this evidence of introgression with a population allele frequency-based analysis, we calculated the four-taxon introgression statistic *f*_d_, which is an extension of the ABBA-BABA D-statistic for population samples [35], in 10-kb windows across the genome for each pair of species involved in putative *BCO2* introgression. The gene trees did not provide evidence of which species within the genera were the likely donor species (Fig 2B and 2C); we therefore chose *Vermivora cyanoptera* and *Leiothlypis ruficapilla ruficapilla* as representatives. *V. cyanoptera* has previously been found to hybridize with *G. formosa* [36], and *L. r. ruficapilla* provided the largest population sample for the *Leiothlypis* introgression analysis. A window inside the *BCO2* gene or within 10 kb of the gene was an outlier exceeding the 99.9th percentile in each of the comparisons except *Leiothlypis–Setophaga ruticilla*, where it exceeded the 99.8th percentile (Fig 3A–3G). The gene tree and *f*_d_ analyses strongly support introgression from *Vermivora* to *G. formosa* and from *Leiothlypis* to *Setophaga* and *Cardellina* at *BCO2*.

Next, to obtain the precise chromosomal locations of the introgressed regions, we examined genotypes in the groups of interest and filtered to retain those fixed or nearly fixed for different alleles between the genera, excluding recipients of gene flow in the filter (Fig 3H and 3I). Evidence of introgression was apparent in a region both upstream and within the *BCO2* gene from *Vermivora* to *G. formosa* and from *Leiothlypis* to *S. ruticilla*, *S. magnolia*, *S. fusca*, *S. petechia*, *S. discolor*, *S. vitellina*, and *C. pusilla* (hereafter, we refer to this group of species as “BISC,” for “*BCO2* introgression in *Setophaga* and *Cardellina*”). *C. rubrifrons*, though it did not fall on the *Leiothlypis* branch of the gene tree (Fig 2A), also showed evidence of introgression in the same region but with fewer sites fixed for *Leiothlypis* alleles (Fig 3I).

We calculated a range of dates for the most recent common ancestor between genera that appeared to have been involved in *BCO2* introgression using the branch lengths from our concatenated UCE tree and three previous estimates of the Parulidae crown age (S3 Fig) [9,37,38]. We estimated that the split between *Leiothlypis* and *Setophaga* and between *Vermivora* and *Geothlypis* occurred between 5.3 and 8.1 Mya (mean 6.7 Mya), and the split between *Setophaga* and *Cardellina* occurred between 2.6 and 4.0 Mya (mean 3.3 Mya). These divergence times are not unprecedented for hybridizing species, including in birds [39,40]. However, adaptive introgression between genera is a rare phenomenon—or at least rarely observed thus far—and further study is needed to evaluate whether these instances in warblers cover unusually great evolutionary distances.

### A unique amino acid substitution is retained in *BCO2* introgression recipient species

Because *BCO2* variants commonly co-occur with carotenoid coloration differences in animals [22–27,41–43] and because the introgression signal at *BCO2* was strong in each comparison we examined (Fig 3A–3G), we hypothesized that the introgressed regions in *Setophaga*, *Cardellina*, and *G. formosa* contained one or several mutations with important implications for the gene’s expression or the protein’s function.

We aligned the *BCO2* coding sequences and amino acid sequences from all species to explore whether possible functional mutations affected the protein itself (S4 Fig). We found no amino acid substitutions common only to *Vermivora* and *G. formosa*. We also found none that were common to only *Leiothlypis* and the BISC species. However, at the third position in exon three, these species had amino acids that were distinct from the rest of the warblers: *Leiothlypis* had an asparagine, and the BISC species had a serine (S4A Fig). Our previous work identified this amino acid as distinct in the *Setophaga* BISC species compared to 68 other bird genomes [25,27]. The nucleotide sequence revealed that a G > T mutation in the third codon position, conferring an asparagine in *Leiothlypis*, was inherited by all of the BISC species, and they all shared a subsequent A > G mutation in the second codon position, conferring a serine (S4B Fig).

In *C. rubrifrons*, we generated higher coverage sequencing data for all five individuals to confirm the allelic makeup of the introgression haplotype. We examined aligned reads and found that in the immediate vicinity of this pair of nucleotides, one individual had two copies of the ancestral *Cardellina* haplotype, one had two copies of the introgression haplotype, and three were heterozygous (S5 Fig). This pattern is consistent with introgression that has not progressed to fixation, but deeper insights into the evolutionary forces driving the pattern in *C. rubrifrons* require additional population sampling.

We cannot say conclusively whether the *Leiothlypis* G > T mutation and/or the subsequent BISC A > G mutation are the functional variant(s) likely to have been under selection following introgression in these species, but we note that they occur in the very small 68-nucleotide stretch at the center of the introgression block where all BISC species have sites fixed for *Leiothlypis* alleles (Fig 3I). The ancestral lysine is large and basic, while the *Leiothlypis* asparagine and the BISC serine are both polar neutral; asparagine is smaller, and serine is much smaller than lysine. We also note that all BISC species show considerable carotenoid-based feather coloration, a phenotype linked in several other birds to reduced *BCO2* expression [25]. Another bright yellow species, *Protonotaria citrea*, had a separate mutation in the first position of the same codon, conferring a glutamic acid (S4 Fig). Glutamic acid is acidic, while the ancestral lysine is basic.

Together, these facts all point to a possible role for the amino acid substitution in disrupting or altering *BCO2* enzyme function or expression in the BISC warblers, leading to increased deposition of feather carotenoids. Alternatively, a nearby mutation in the noncoding sequence could alter *BCO2* expression. In the 68-nt sequence described above that was common to all BISC species, there were three SNPs in the intron immediately before exon 3 that fit the same inheritance pattern as the amino acid substitution. One of these SNPs could affect *BCO2* expression. In a study identifying *BCO2* introgression underlying yellow plumage in manakins, Lim and colleagues [44] found that the highest concentration of introgressed alleles fell in the same intron, which could suggest a conserved role for this region in harboring transcription factor binding sites or other regulatory sequences that control when or where the gene is expressed.

Whether or not the serine substitution is the functional change under selection, the lysine-to-arginine-to-serine sequence of mutations can help inform a preliminary hypothesis of how *BCO2* gene flow occurred in Parulidae. The inheritance pattern strongly suggests that there was a single introgression event from *Leiothlypis* into one of the BISC species; the asparagine-to-serine mutation occurred in that species; and the new *BCO2* sequence was subsequently spread to the rest of the species in the group. Because *Setophaga discolor* and *S. vitellina* share very similar introgression haplotypes (Fig 3I), and because *S. vitellina* most likely originated as an isolated island population of *S. discolor* [45], the initial introgression event from *Leiothlypis* to the BISC species probably predates the isolation of *S. vitellina* on the Cayman and Swann Islands. However, it most likely occurred, at a minimum, after the split between *S. ruticilla* and *S. citrina*. From previous estimates of the Parulidae crown age [9,37,38] and branch lengths in our concatenated phylogeny, we calculated that the initial introgression from *Leiothlypis* occurred between 532 thousand and 1.9 million years ago (mean 679 kya–1.6 Mya). The donor species may have been the ancestor of several extant *Leiothlypis* species, including *L. celata*, *L. crissalis*, *L. ruficapilla*, *L. virginiae*, and *L. luciae*, which we estimated to have diverged between 615 and 984 kya (mean 785 kya). These dates are summarized in S3 Fig.

The precise order and timing of subsequent introgression events among the BISC species are less clear. If one BISC species received initial *BCO2* gene flow from *Leiothlypis* and subsequently spread the haplotype to each other species in the group, the first species’ haplotype would result from one instance of introgression, while the rest would result from two. Alternatively, the haplotype may have spread several times sequentially from species to species, resulting in third-order or higher introgression haplotypes (S6A Fig). Extrapolating from the model of Veller and colleagues [46] in which introgressed DNA is rapidly purged from the recipient genome and then levels off, we would predict second- or higher-order introgression events would result in progressively smaller introgression haplotypes in the recipient species (S6B and S6C Fig). With this simple model as a guide, *S. magnolia* would be the likeliest recipient of the original *Leiothlypis* introgression, since it has the largest remaining introgression haplotype (Fig 3I). However, each species may have different demographic histories, which would render the effects of drift more or less important. Additional population sampling among these species is needed to illuminate the order and direction of historical *BCO2* introgression.

### Introgression of carotenoid dehydrogenase *BDH1L* and carotenoid ketolase *CYP2J19* point to a possible single origin of red plumage in warblers

Research into the genetic basis of red coloration in birds has implicated the combined action of two genes, *BDH1L* and *CYP2J19*, in the conversion of yellow dietary carotenoids to red ketocarotenoids [28,29]. Though many warblers show carotenoid pigmentation, only seven species are believed or known to deposit ketocarotenoids in feathers [47,48], including two species of *Setophaga*, three species of *Cardellina*, and two species of *Myioborus*. The former two have orange plumage, and the latter five are red. The five red species predominantly occur in Central America—or, in the case of *Myioborus miniatus*, the red subspecies are in Central America—which raises the question of whether their similar distributions reflect a history of red plumage introgression between two or more of the species.

**Fig 4:**
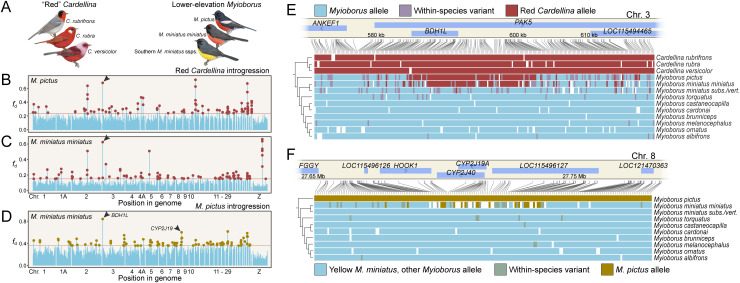
*BDH1L* and *CYP2J19* introgression in red warblers. **(A)** Images of the species involved, including red *Cardellina* species and *Myioborus pictus*, and *M. miniatus* subspecies. *M. m. miniatus* occurs in Mexico, and *M. m. verticalis* and *subsimilis* occur across much of South America. Other subspecies exist but were not sequenced. **(B–D)** Introgression metric *f*_d_ calculated in 10-kb windows. (B and C) *Cardellina rubra* as the presumed donor and (B) *M. pictus* and (C) *M. m. miniatus* as the presumed recipients. (D) *M. pictus* as the presumed donor species and *M. m. miniatus* as the recipient. Black arrows indicate where windows within or adjacent to *BDH1L* or *CYP2J19* are among 99.9% outliers. **(E and F)** Genotypes at biallelic SNPs in the (E) *BDH1L* region, filtered to retain sites fixed for one allele in red *Cardellina* species and with ≥ 90% frequency of a different allele in yellow *Myioborus* species and (F) *CYP2J19* region, filtered to retain sites fixed for one allele in *M. pictus* and both fixed for a different allele in yellow *M. miniatus* and with ≥90% frequency of the yellow allele in the other yellow *Myioborus* species. Warbler illustrations courtesy of Cornell Lab of Ornithology, illustrated by David Quinn, from Birds of the World (https://doi.org/10.2173/bow). The data and code needed to generate this figure can be found at https://doi.org/10.5061/dryad.1zcrjdg3v and from NCBI at BioProject PRJNA630247.

To test for evidence of gene flow at *BDH1L* and *CYP2J19* in warblers, we compared the gene trees to the warbler species tree. On the gene tree derived from an 11-kb region within and upstream of *BDH1L* that contained 977 SNPs, *M. pictus* and *M. miniatus miniatus*, both of which have bright red underparts, fell on the branch of the tree with the red *Cardellina* species, *C. rubrifrons*, *C. rubra*, and *C. versicolor* (S7A Fig), consistent with gene flow from *Cardellina* to *M. pictus* and *M. m. miniatus*. In the genomes of both *M. pictus* and *M. m. miniatus*, a 10-kb window overlapping *BDH1L* was among the highest outliers in *f*_d_, indicating a strong signal of introgression from *Cardellina* (Fig 4B and 4C). Finally, genotypes across the gene region, filtered for sites with fixed or nearly fixed differences between red *Cardellina* and yellow *Myioborus* species, showed clear regions of *Cardellina* ancestry in both *M. pictus* and *M. m. miniatus* within and upstream of *BDH1L* (Fig 4E). The major fixed region in the *M. pictus* introgressed haplotype encompassed the entire major fixed introgressed region in *M. m. miniatus* and was roughly 2 kb larger. We conclude that the most likely scenario is one in which a portion of the *BDH1L* gene introgressed from a red *Cardellina* species into *M. pictus* and subsequently introgressed from *M. pictus* into *M. m. miniatus*. We estimated that *Cardellina* and *Myioborus* diverged between 2.6 and 4.0 million years ago (mean 3.3 Mya) (S3 Fig).

The gene tree derived from a 37-kb region within and upstream of *CYP2J19* that contained 2657 SNPs did not show evidence of gene flow between *Cardellina* and *Myioborus*, but *M. m. miniatus*, which has red underparts, was sister to *M. pictus*, which also has red underparts, rather than *M. m. subsimilis* and *M. m. verticalis*, which both have yellow underparts (S7B Fig). This pattern is consistent with gene flow between *M. pictus* and *M. m. miniatus* at *CYP2J19*. Three windows surrounding *CYP2J19* were *f*_d_ outliers exceeding the 99.9th percentile in *M. m. miniatus* (Fig 4D). We also examined genotypes across the gene region, filtered for sites where *M. pictus* had a different allele from yellow *M. miniatus* and 90% of other *Myioborus* species’ alleles. *M. m. miniatus* had more *M. pictus* alleles within and just outside the *CYP2J19* gene than in flanking regions (Fig 4F). These results are all consistent with gene flow from *M. pictus* to *M. m. miniatus* at *CYP2J19*.

Together, these results suggest that the red underparts of several *M. miniatus* subspecies, including *M. m. miniatus*, were likely acquired through gene flow from *M. pictus*, and that the red underparts of *M. pictus* were either acquired or enhanced through gene flow from *Cardellina*. The geographic pattern of plumage color in *M. miniatus* supports this idea, as red-bellied subspecies are found in northern Central America—close to and overlapping with the *M. pictus* range—while yellow-bellied subspecies are found in South America, with orange-bellied subspecies in the middle [49]. In at least one instance, hybrid offspring of *M. pictus* and *M. miniatus* have been produced in Arizona, USA (https://macaulaylibrary.org/asset/31405941). In addition, the distribution of *M. pictus* overlaps with all three red *Cardellina* species in the USA and Central America. While red plumage was already known to be rare among parulid warblers, these findings raise the possibility that its occurrence in the family has a single evolutionary origin in *Cardellina*.

### Genome-wide signatures of gene flow

The above analyses pointed to particular instances of gene introgression between warbler species. We next asked whether these introgression events were indicative of greater genome-wide gene flow between these species, or whether there were distinct broad gene flow patterns among the warbler genera. To address this, we calculated the D-statistic and f4-ratio, which estimates the admixture proportion in the genome between two taxa [50], for pairwise comparisons of the species involved in the introgression events we identified above and several other species in the same genera (S8 Fig). In some cases, species involved in color gene introgression did show a greater signal of hybridization, including red *Cardellina* with *M. pictus*, *S. fusca* with *S. magnolia*, and *S. petechia* with *S. discolor*. But in most cases, including *Leiothlypis*–*Setophaga* comparisons, *Setophaga*–*Cardellina* comparisons, and *Geothlypis*–*Vermivora* comparisons, the species sharing color genes as uncovered in this study did not show greater hybridization signals genome-wide than those not sharing color genes.

Because many of these pairings were between distantly related species (S3 Fig), hybridization between them was probably quite rare even when the color gene introgression occurred, and donor DNA was efficiently purged from the recipient genome. The fact that a signal of *Cardellina*–*M. pictus* hybridization is clearly visible in this analysis may reflect the more recent *Cardellina*–*Myioborus* divergence, which we estimate to be 2.61–4.02 Mya (mean 3.33 Mya) (S3 Fig), or it may suggest that *BDH1L* introgression is recent, and *Cardellina* DNA is still being purged from the *M. pictus* genome.

### Introgression, sexual selection, and diversification

In many instances of adaptive introgression, the selective mechanism is clear. Introgression of wing patterns in *Heliconius* butterflies facilitates their mimicry [51]; introgression of resistance genes in mice protects them from the rodent poison warfarin [52]. In the cases we have identified in warblers, the most likely traits spreading via introgression are yellow and red plumage coloration, and the most likely driver of their introgression is sexual selection. These cases add substantially to known examples of apparent adaptive introgression likely driven by sexual selection. Most previous studies that discovered similar situations involved traits that had only partially introgressed into recipient populations [31,53–56], rather than those that introgressed completely and became fixed in recipients (but see Lim and colleagues [44]). The latter case may be as common or more so than the former, but only discoverable by looking for evidence of past hybridization rather than active hybrid zones. The fact that colorful plumage appears to have spread many times among warbler species raises the question of whether some color phenotypes are or were universally attractive across the family. Under this idea, once one species acquired the trait as an evolutionary novelty, it would eventually spread to relatives, as long as there was at least some interbreeding among them. Such a preference could arise through several evolutionary processes, including perceptual bias [57] or “honest signaling” [58]. As the genetics underlying sexual traits continue to be uncovered and hybridization research continues to expand beyond contemporary hybrid zones, similar situations to that in the warblers will likely be discovered, especially among animals where color signaling plays a prominent role in sexual selection.

In contrast to our finding that carotenoid-related genes have frequently introgressed between warbler species, previous studies have found that despite a small suite of genes often underlying traits related to melanism, these genes seem to repeatedly evolve in parallel to produce melanistic phenotypes rather than spread via introgression. For example, Baiz and colleagues [27] found no evidence of gene flow on the *ASIP* gene tree, even though *ASIP* divergence was associated with plumage differences among several pairs of warbler species. Selection on separate melanin genes or separate regulatory regions controlling the same gene have also been implicated in color divergence in Solomon Islands flycatchers and a radiation of South American seedeaters, respectively [59,60]. This trend requires more research to confirm, but it suggests that the evolutionary “hurdles” presented by altering carotenoid pathways are more difficult to solve than those related to melanin pathways and, therefore, more likely to be “borrowed” from a related species.

Our finding that carotenoid genes seem to readily spread between warbler species has potential implications for the group’s diversification. If the phenomenon increases the likelihood of interbreeding between species, it has the potential to counteract forces leading to speciation, at least if the species are in the early stages of divergence. Our analyses here had no ability to detect introgression between sister species, so we cannot say definitively whether this process occurred in warblers. On the other hand, if one species or population changed color due to introgression from a distant relative, this might introduce a new premating barrier with any close relatives who used color to select mates, thereby accelerating speciation and contributing to diversification. Past studies have found that hybridization can, in fact, contribute to diversification, e.g., by providing new genomic substrates for adaptation in African cichlids [6] and Hawaiian silverswords [61]. The parulid warblers may be a fruitful system to continue this area of research or even extend it: much of their radiation appears to have been characterized by divergence in sexual traits at least as much as divergence of ecological niches [62], and recent evidence from birds of paradise suggests intergeneric hybridization may not be a rare phenomenon in such a group [40,63].

Parulid warblers have been noted to produce intergeneric hybrids and more generally hybridize outside of hybrid zones fairly frequently [16,17]. The present study suggests that such crosses could have played a major role in producing the pattern of plumage color variation that makes the family so distinctive. We present evidence from three genes that can have large effects on carotenoid-based phenotypes. Similar processes may have played out across the genome, but because genome-wide signals of hybridization may not point to these events, a targeted approach appears to be necessary to detect them.

## Methods

### Data collection and sequencing

Samples for DNA extraction and sequencing were obtained from a combination of frozen museum tissues, blood in lysis buffer collected from mist-netted birds in the field, and, in the case of extinct *Vermivora bachmanii*, toepads cut from museum specimens (S1 Table). All samples collected for this study were obtained under a protocol approved by IACUC at Cornell University (protocol no. 2015-0065) or Penn State University (protocol no. 201900879) and under a USGS federal master bird banding permit (24043 to D.P.L.T.), as well as the New York State Department of Environmental Conservation (banding permit no. 156), the Pennsylvania Game Commission (banding permit 46121), and the Pennsylvania State Forest (SFRA permit 2220). DNA was extracted using Qiagen DNeasy Blood and Tissue kits. We used 2 mm^3^ of tissue and 75 μL of blood as inputs for digestion. The sequencing library preparation procedure for *Setophaga*, *Catharopeza*, *V. cyanoptera*, and *V. chrysoptera* DNA is outlined in [27]. The procedure for *V. bachmanii* is described in [64]. For all other samples, paired-end libraries were prepared using Illumina DNA Prep kits and dual-indexed, with a 350-bp insert size targeted. Libraries were sequenced across seven lanes of an Illumina NextSeq 2000. Reads were trimmed using AdapterRemoval [65] and aligned to the *S. coronata* reference genome using Bowtie 2 [66], and PCR duplicates were marked with PicardTools. The final average coverage across all samples was 3.79×. We evaluated whether mapping rates were uneven across the warbler tree, which could bias results that rely on phylogenetic relationships [67,68]. Mapping rates were obtained from bowtie log files. We found very consistent mapping rates across the tree (S9 Fig), with only a few species showing reduced mapping, and these species were not clustered in the tree.

### Phylogenetic analysis

We only required a single sequence for each species for phylogeny-building, so to take advantage of replicate individuals per species and increase confidence in base calling via higher coverage, we combined alignment BAM files for all individuals of each species to make species-BAM files. Next, we made a master variant call format (VCF) file by running bcftools mpileup and call commands [69] with a ploidy of 2, then filtered to remove low-quality SNPs (QUAL < 10) and set low-quality genotypes (GQ < 10) to uncalled using bcftools. Finally, we filtered genotypes by read depth using bcftools. Since some species’ data varied in the number of individuals they contained, we customized read depth filtering for each species after examining depth distributions across a reduced dataset. Minimum read depth permitted ranged from 2 to 5; maximum depth permitted ranged from 20 to 125. The code with precise cutoffs for all species is available from Dryad [70].

We next extracted UCE loci and flanking sequences for each species with a custom script that used bcftools view to subset the VCF file to the region of interest, then samtools faidx [69] and bcftools consensus to extract each species’ sequence, incorporating variants from the VCF. Detailed methods for identifying UCE loci and flanking sequences in the *S. coronata* reference genome are described in [27]. Briefly, the Tetrapods-UCE-5kv1 probe set [71] was aligned to the *S. coronata* reference; only uniquely aligning sequences were retained; loci were thinned to 3 kb separation distances; and 1 kb of flanking sequence on each side was included in final fasta files for 2-kb total lengths. We aligned and trimmed the species UCE sequences using mafft [72] and GBlocks [73] implemented in PHYLUCE [74]. No UCEs were missing for any species, so we retained all 4016 loci.

We used two different methods for tree building: concatenation and species tree estimation from gene trees. For the former, we concatenated all 4016 loci and used the resulting alignment as the input to IQ-TREE [75]. We first repeated all of the above steps with just 25 species (for computational tractability) and ran the model finder function in IQ-TREE. The best supported DNA evolution model was TVM + F + I + R3, so we used that model when running the program with the full dataset. The final run also included 1,000 replicates of an approximate likelihood ratio test (aLRT) to compute branch support.

Separately, we used the individual aligned, trimmed UCE loci as inputs to runs of IQ-TREE using the same DNA evolution model as the full dataset and 1,000 aLRT replicates per tree. The resulting 4,016 trees were concatenated and used as inputs for weighted TREE-QMC, a quartet-based method for species tree estimation from gene trees [76]. Because this method does not estimate terminal branch lengths, we finally re-ran the concatenated tree procedure in IQ-TREE described above, but constrained to the TREE-QMC topology. A comparison between the two trees is displayed in S1 Fig.

In downstream analyses that required a species tree, we used the concatenated tree with estimated branch lengths from IQ-TREE. To display the phylogeny as an ultrametric tree in Fig 1, we first rooted the tree on *Icteria virens*, dropped the *I. virens* tip, then ran the chronos function from the package ape [77] in R [78] with the “clock” model of substitution rate variation among branches and a lambda of one.

To examine the effect of combining individuals of the same species together into a single BAM file before variant calling, we made a tree following the same procedure but with three individuals per species and one species per genus (with the exception of *Catharopeza*, for which we only had two samples). We subset the resulting tree to one branch per genus and compared relative node ages to our final concatenated UCE tree subset to the same taxa. Older nodes were at similar positions between the two trees, but the separate-individual tree’s younger nodes were comparatively older than the combined-individual tree (S10 Fig). Because we did not do further analyses to evaluate our tree-building method, we cannot say which method is superior. However, our interpretations of gene tree topologies did not rely on branch lengths, and windowed *f*_d_ analyses used separate individual datasets. The study’s conclusions were therefore unaffected by this methodological choice.

### Color gene introgression analyses

To test for evidence of gene flow among species at key genes related to coloration, we compared the UCE species tree to gene trees for *BCO2*, *CYP2J19*, and *BDH1L*. We first generated gene annotations for the *S. coronata* reference genome from Zebra Finch assembly (accession GCF_003957565.2) using LiftOff with default options [79]. Gene sequences were then extracted using bcftools consensus as described above for UCE loci, then aligned with mafft. Trees were made using IQ-TREE, and in each case, we used the best supported DNA substitution model as determined by the model finder. We reduced the regions of interest to roughly 1.8-kb, 10.9-kb, and 36.9-kb stretches of DNA based on the largest mostly contiguous sequences that made up apparent introgression haplotypes for *BCO2*, *BDH1L*, and *CYP2J19*, respectively (S11 Fig). For *CYP2J19* and *BDH1L*, after viewing the gene trees with all species included, we created a new version of the master VCF file described above for just the genomic regions of interest in *Cardellina*, *Myioborus*, and *B. lachrymosus* as an outgroup, separating the red-breasted *M. miniatus* samples (subspecies *miniatus*) from the yellow-breasted samples (subspecies *subsimilis* and *verticalis*). This was done to avoid redoing the computationally intensive step of calling variants across the entire genome for all species. Filtering for the two subspecies followed the filtering scheme described above, with read depth filtering cutoffs chosen based on the range of cutoffs for similar numbers of input individuals in the full dataset.

In several instances where species relationships on a gene tree did not match the species tree and were thus suggestive of gene flow, we ran windowed analyses calculating the introgression statistic *f*_d_ [35], an extension of the ABBA-BABA D-statistic [80] which uses population samples to estimate the proportion of a genomic window shared between species due to introgression. To run these analyses, we first called variants from individual BAM files of species of interest (in contrast to the species-combined BAM files described above). Although we were most interested in introgression at the genes themselves, we ran these analyses across the entire genome in 10-kb windows to put introgression around the gene of interest in the context of genome-wide introgression patterns. In the case of *BCO2*, we ran a version with *V. cyanoptera* as the “donor” (i.e., P3 from Martin and colleagues [35]) and *G. formosa* as the “recipient” (i.e., P2 from Martin and colleagues [35]) and a series with *L. ruficapilla* as the donor and *S. ruticilla*, *S. magnolia*, *S. fusca*, *S. petechia*, *S. discolor*, and *C. pusilla* as the recipients. For *CYP2J19*, we used *M. pictus* as the donor and *M. m. miniatus* as the recipient. For *BDH1L*, we used *C. rubra* as the donor and *M. pictus* and *M. m. miniatus* separately as the recipients. The presumed donor versus recipient taxa can be determined from the topology of the gene tree: the recipient taxon will fall on the branch of the tree normally occupied by the donor taxon. For example, in Fig 2C, *G. formosa* is the recipient, and a *Vermivora* species is the donor. This is because the sequence of the gene will have followed the evolutionary trajectory of the donor species before subsequently moving into the recipient species via gene flow.

We sought to estimate the divergence times between genera that showed evidence of color gene exchange. Because we did not time-calibrate the phylogeny we generated, we used a combination of our phylogeny and previous time-calibrated trees to estimate divergence times of interest. We used the warbler crown age from Barker and colleagues [37], Oliveros and colleagues [9], and Claramunt and colleagues [38], and branch lengths from our phylogeny to calculate a range of divergence times, displayed in S3 Fig.

To more precisely determine the locations of introgressed DNA in the genome, we filtered the master VCF file based on several subsets of species genotype data and visualized the resulting variants using IGV [81]. We filtered each data subset to retain sites where signatures of introgression would be most apparent: sites that were fixed or nearly so for different alleles between two lineages of interest. In each case, we used trial-and-error to adjust allele frequency and missingness thresholds to omit sites not fitting this pattern but retain as many others as possible. In each case below, we retained only biallelic SNPs. For *Vermivora*–*Geothlypis BCO2* introgression, we subset the data to sites where *Vermivora* species were fixed for one allele and *Geothlypis*, excluding *G. formosa*, had ≥90% frequency of the other allele. For *Leiothlypis*–BISC species *BCO2* introgression, we included only sites where *Leiothlypis* had ≥90% frequency of one allele and *Setophaga* (excluding the BISC species) and *Myioborus* both had ≥90% frequency of the other allele. We included *Myioborus* in the filter rather than *Cardellina* due to uncertainty over which *Cardellina* species received *BCO2* introgression. In both *BCO2* genotype subsets, we allowed <10% missing genotypes. For *BDH1L* introgression, we included sites where the three red *Cardellina* species were fixed for one allele and yellow *Myioborus* species, including yellow *M. miniatus* subspecies, had ≥90% frequency of a different allele. We allowed for 40% missing genotypes in red *Cardellina* and 30% missing genotypes in yellow *Myioborus*. For *CYP2J19* introgression, we included only sites where *M. pictus* was fixed for one allele, yellow *M. miniatus* was fixed for the other allele, and the rest of *Myioborus*, excluding red *M. miniatus*, had ≥90% frequency of the yellow *M. miniatus* allele. No missing data was allowed for *M. pictus* or yellow *M. miniatus*. We allowed <30% missing genotypes in the rest of *Myioborus*, excluding red *M. miniatus*.

In order to better understand potential functional changes to *BCO2* that may be related its introgression between species, we generated a coding sequence alignment for the gene using the gene alignment described above, manually extracting the exons based on the gene annotation and translating to an amino acid alignment in Geneious Prime 2021.2.2. To display the alignment in S4 Fig, we chose species that comprised most of the variation shared between any two species from our genera of interest in the gene’s first two exons. We noted that the nucleotide of greatest interest, which conferred a serine in the BISC species, was uncalled in *S. vitellina*, rather than called a G as in the rest of the BISC species. Viewing the individual BAM files revealed that, though there was low coverage in the region, raw reads only contained a G at the nucleotide in question, and none contained the presumed ancestral A (S12 Fig). As a result, in S4 Fig we display the *S. vitellina* nucleotide sequence with a G and the amino acid sequence with a serine. Similarly, at the first position of the same codon, the *P. citrea* genotype was uncalled, but 18 out of 19 reads at the position show a G rather than the presumed ancestral A (S13 Fig). Therefore, we display the *P. citrea* nucleotide sequence with a G and the amino acid sequence with a glutamic acid in S4 Fig.

### Genome-wide signatures of gene flow

To evaluate genome-wide signals of gene flow, we calculated the D-statistic and f4-ratio for species pairs discovered to exchange color genes in the previous analyses and several relatives from the same genera as comparisons. We first called variants from individual BAM files using bcftools, setting a minimum minor allele count of 2 and setting to uncalled any genotype with a genotype quality of less than 10. Three variant files were created: one for *Leiothlypis*–*Setophaga*–*Cardellina* comparisons, one for *Geothlypis*–*Vermivora* comparisons, and one for *Cardellina*–*Myioborus* comparisons. We then ran the command dtrios from DSuite [82], providing the species topology and using 200 jackknife blocks across the genome. To display the results, we used a custom R script to select the P1 species resulting in the maximum D and f4 values for each comparison and transform the results into a matrix.

## Supporting information

S1 Table Information on provenance and tissue type for all samples sequenced for or used in the study. (XLSX)

S1 Fig Differences between concatenated and quartet-based gene tree reconciliation methods for tree estimation. Nodes or groups that differ between methods are highlighted. The data and code needed to generate this figure can be found at https://doi.org/10.5061/dryad.1zcrjdg3v and from NCBI at BioProject PRJNA630247.
(PNG)

S2 Fig *BCO2* gene tree with branch support. Support values were calculated by 1,000 replicates of an approximate likelihood ratio test (aLRT). The data and code needed to generate this figure can be found at https://doi.org/10.5061/dryad.1zcrjdg3v and from NCBI at BioProject PRJNA630247.
(PNG)

S3 Fig Concatenated ultra-conserved element warbler phylogeny with estimated divergence times relevant to introgression timing and relationships between hybridizing species. Intergeneric hybridization occurred between *Leiothlypis* and *Setophaga* (node 1), *Setophaga* and *Cardellina* (node 2), *Vermivora* and *Geothlypis* (node 1), and *Cardellina* and *Myioborus* (node 3). Introgression also occurred between *Myioborus pictus* and *M. miniatus* (node 4). *BCO2* introgression from *Leiothlypis* to *Setophaga* likely occurred between nodes 5 and 7. Node 6 indicates that an ancestor of most extant *Leiothlypis* species may have been the gene flow donor. The data and code needed to generate this figure can be found at https://doi.org/10.5061/dryad.1zcrjdg3v and from NCBI at BioProject PRJNA630247.
(PNG)

S4 Fig *BCO2* coding sequence alignment. **(A)** *BCO2* amino acid alignment of species involved in introgression and several relatives, showing the second and third exons. No amino acids are either unique to *Vermivora* and *Geothlypis formosa* or unique to *Leiothlypis* and the introgressed *Setophaga* and *Cardellina* species. Within-species variation is shown as an X. The sequence corresponding to the human mitochondrial targeting sequence (MTS) is indicated with a blue bar. **(B)** Alignment of the *BCO2* coding sequence and its translation at the start of the third exon. The codon of interest is the third in the exon. Introgressed species likely inherited asparagine from *Leiothlypis*, which was subsequently replaced by serine after an A > G mutation in the second codon position.
(PNG)

S5 Fig Sequencing reads from each of five *Cardellina rubrifrons* individuals aligned to the *Setophaga coronata* reference in part of the *BCO2* introgression region. With the exception of the alternate G (versus the reference A) at position 1,587,170, denoted by a black arrow, each variant shown differs between the ancestral *Cardellina* haplotype and the introgression haplotype. Homozygote versus heterozygote status is noted for each individual. Red arrows denote the two SNPs that confer an asparagine in *Leiothlypis* and a serine in the *BCO2* introgression recipient species (S4B Fig). Figure adapted from IGV [81].
(PNG)

S6 Fig Schematic showing the fate of DNA from an original “donor” species under different introgression scenarios involving multiple recipient species. **(A)** Summary of three scenarios in which introgression proceeds from a donor species into three recipient species (i) in three independent gene flow events, (ii) first to one recipient and then from that recipient into two “second-order” recipients in two independent gene flow events, (iii) in three successive introgression events from one recipient to the next. The table shows how many introgression events would result in the introgression haplotype present in each species under each scenario. **(B, C)** The proportion of original donor DNA predicted to be present in recipient species under scenarios (ii) and (iii), based on the model from Veller and colleagues [main text citation 46]. These schematics show how new pulses of recombination are expected to reduce the size of the original introgression haplotype in second-order and higher introgression events.
(PNG)

S7 Fig “Gene” trees for genes related to red coloration. **(A)** Tree estimated from a 10.9 kb region of chr. 3 within and upstream of *BDH1L*. **(B)** Tree estimated from a 36.9 kb region of chr. 8 within and upstream of *CYP2J19*. Support values were calculated by 1,000 replicates of an approximate likelihood ratio test (aLRT). The data and code needed to generate this figure can be found at https://doi.org/10.5061/dryad.1zcrjdg3v and from NCBI at BioProject PRJNA630247.
(PNG)

S8 Fig Genome-wide gene flow metrics among genera sharing plumage genes. The D-statistic, above the diagonal in red, indicates a signal of shared alleles between two taxa. The f4-ratio, below the diagonal in blue, indicates the proportion of admixture in the genome between two taxa in the genome. (A) *Leiothlypis*, *Setophaga*, and *Cardellina*, including the species suspected to be involved in *BCO2* introgression and several relatives. (B) *Vermivora* and *Geothlypis*. (C) *Cardellina* and *Myioborus*. The data and code needed to generate this figure can be found at https://doi.org/10.5061/dryad.1zcrjdg3v and from NCBI at BioProject PRJNA630247.
(PNG)

S9 Fig Mapping alignment rates of each species’ raw reads to the *Setophaga coronata* reference genome. Error bars are standard errors. There is no reference bias or phylogenetic signal apparent in the alignment rates, which might be observed if species more diverged from the reference (*S. coronata*, denoted with a black arrow) showed lower alignment rates. The data and code needed to generate this figure can be found at https://doi.org/10.5061/dryad.1zcrjdg3v and from NCBI at BioProject PRJNA630247.
(PNG)

S10 Fig Evaluating the potential influence of different variant-calling methods on tree-building. **(A)** The final concatenated ultra-conserved element (UCE) tree from main text Fig 1 subset to one species per genus. **(B)** A concatenated UCE tree made using three individuals per species from one species per genus. **(C)** Comparison of relative node ages between the two trees. Ages are fairly similar, but younger nodes are comparatively older in the combined-individuals tree. The data and code needed to generate this figure can be found at https://doi.org/10.5061/dryad.1zcrjdg3v and from NCBI at BioProject PRJNA630247.
(PNG)

S11 Fig Genotype plots from main text Figs 3 and 4 indicating which regions were selected for gene tree building. **(A)** *BCO2*, **(B)** *BDH1L*, **(C)** *CYP2J19*. The data and code needed to generate this figure can be found at https://doi.org/10.5061/dryad.1zcrjdg3v and from NCBI at BioProject PRJNA630247.
(PNG)

S12 Fig Reads from both *Setophaga vitellina* individuals showing the sequenced nucleotide at the position of interest in the *BCO2* third exon from Main Text Fig 4B. Although the genotype was uncalled in the master VCF file, every read at the position contained the allele from the BISC introgression haplotype. Because the gene is in reverse orientation in the *S. coronata* assembly, it is shown here as C, rather than G. Figure adapted from IGV [81].
(PNG)

S13 Fig Reads from all *Protonotaria citrea* individuals showing the sequenced nucleotide at the first position of the codon of interest in exon 3, highlighted in Main Text Fig 4B. The nucleotide was uncalled in the master VCF file for *P. citrea*, but only one out of 19 total reads show any nucleotide other than a G. Because the gene is in reverse orientation in the *Setophaga coronata* assembly, it is shown here as C, rather than G. Figure adapted from IGV [81].
(PNG)

## Abbreviations

aLRT: approximate likelihood ratio test
VCF: variant call format
UCE: ultra-conserved element
