# Supplementary figures and images for: A colorful legacy of hybridization in wood-warblers includes frequent sharing of carotenoid genes among species and genera
Source: PLoS Biol. 2025 Dec 11;23(12):e3003501. doi: 10.1371/journal.pbio.3003501 (PMC12698001; doi:10.1371/journal.pbio.3003501)

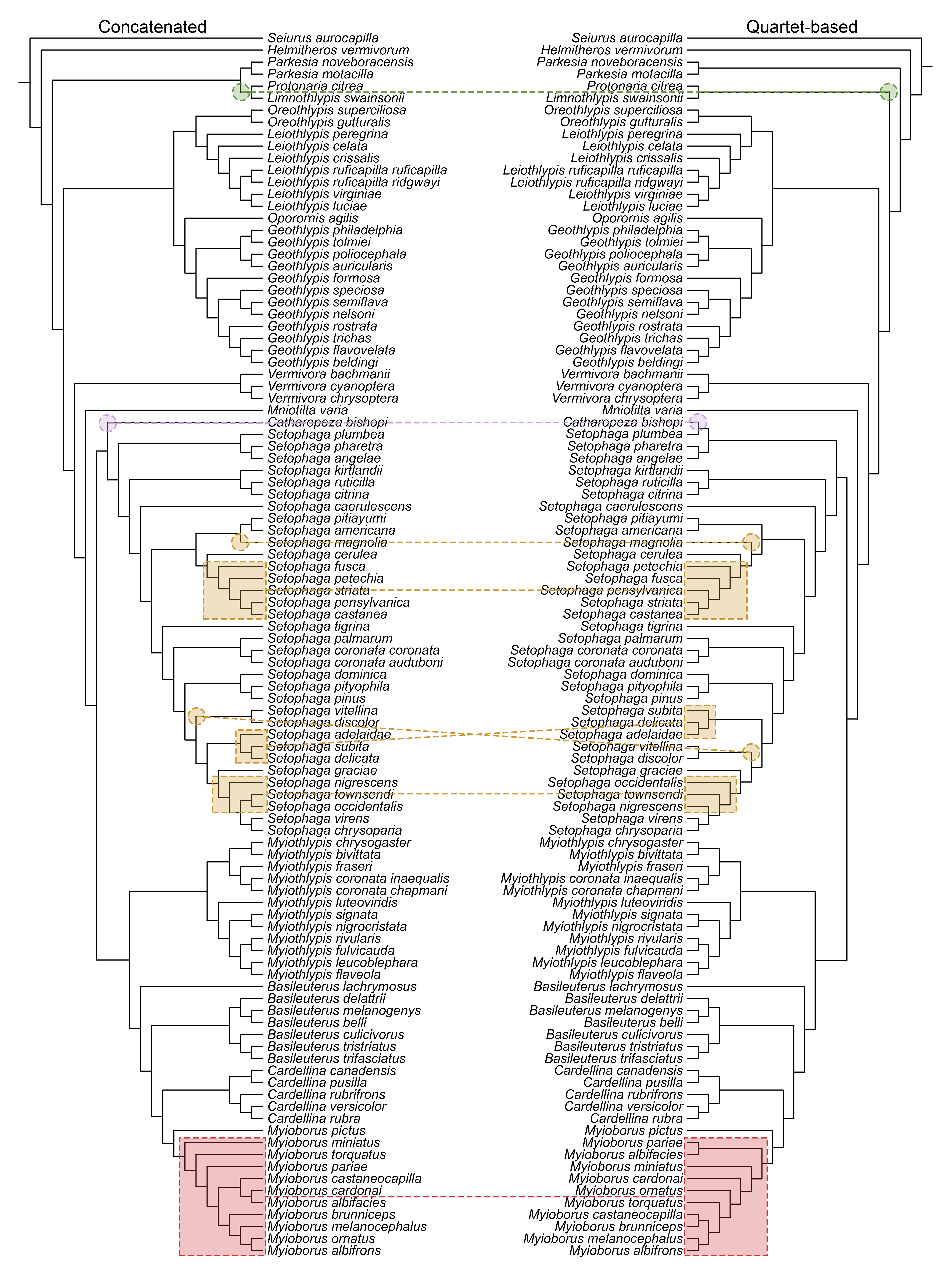

Supplement: S1 Fig — Nodes or groups that differ between methods are highlighted. The data and code needed to generate this figure can be found at https://doi.org/10.5061/dryad.1zcrjdg3v and from NCBI at BioProject PRJNA630247. (PNG) [file pbio.3003501.s002.png]

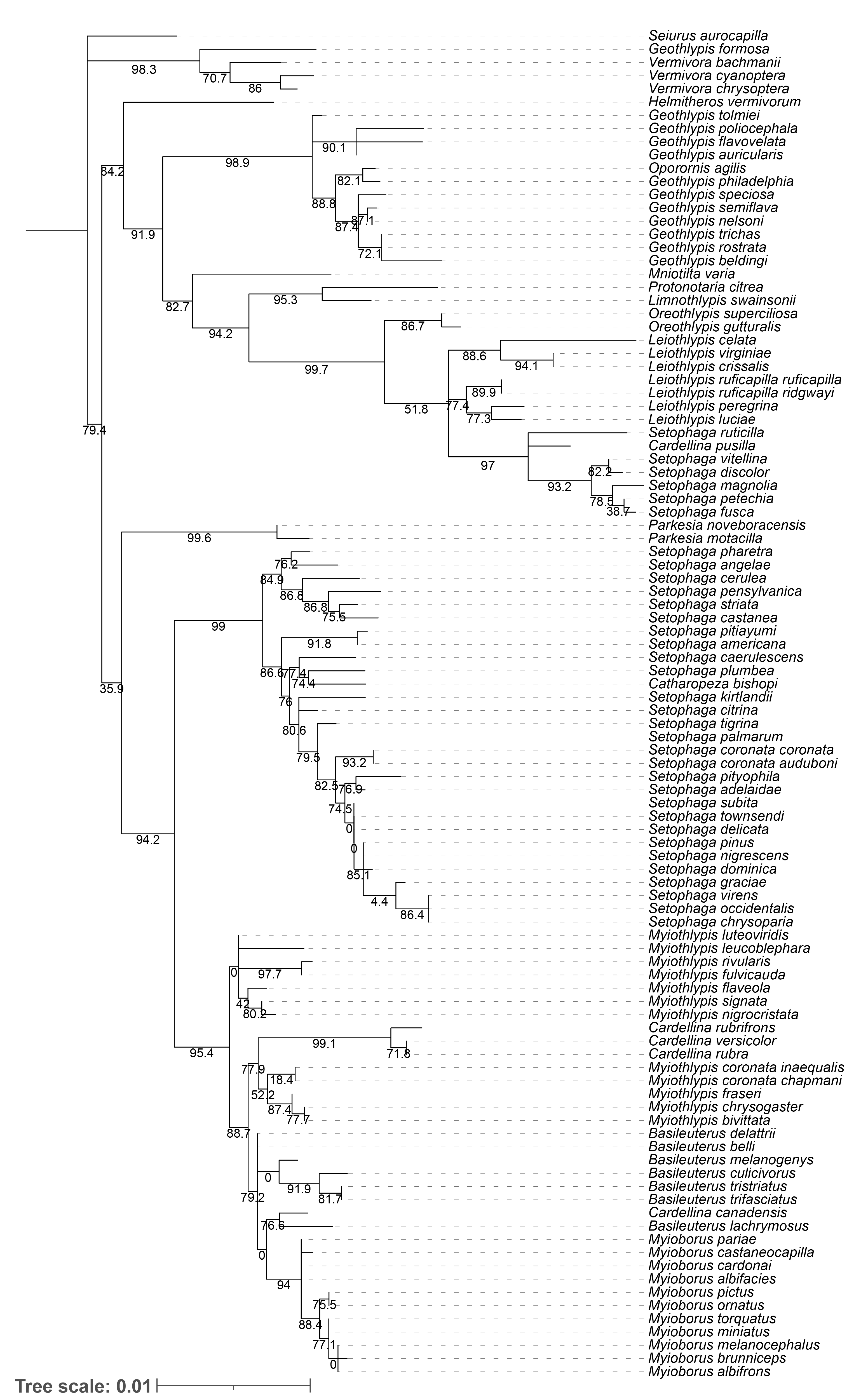

Supplement: S2 Fig — Support values were calculated by 1,000 replicates of an approximate likelihood ratio test (aLRT). The data and code needed to generate this figure can be found at https://doi.org/10.5061/dryad.1zcrjdg3v and from NCBI at BioProject PRJNA630247. (PNG) [file pbio.3003501.s003.png]

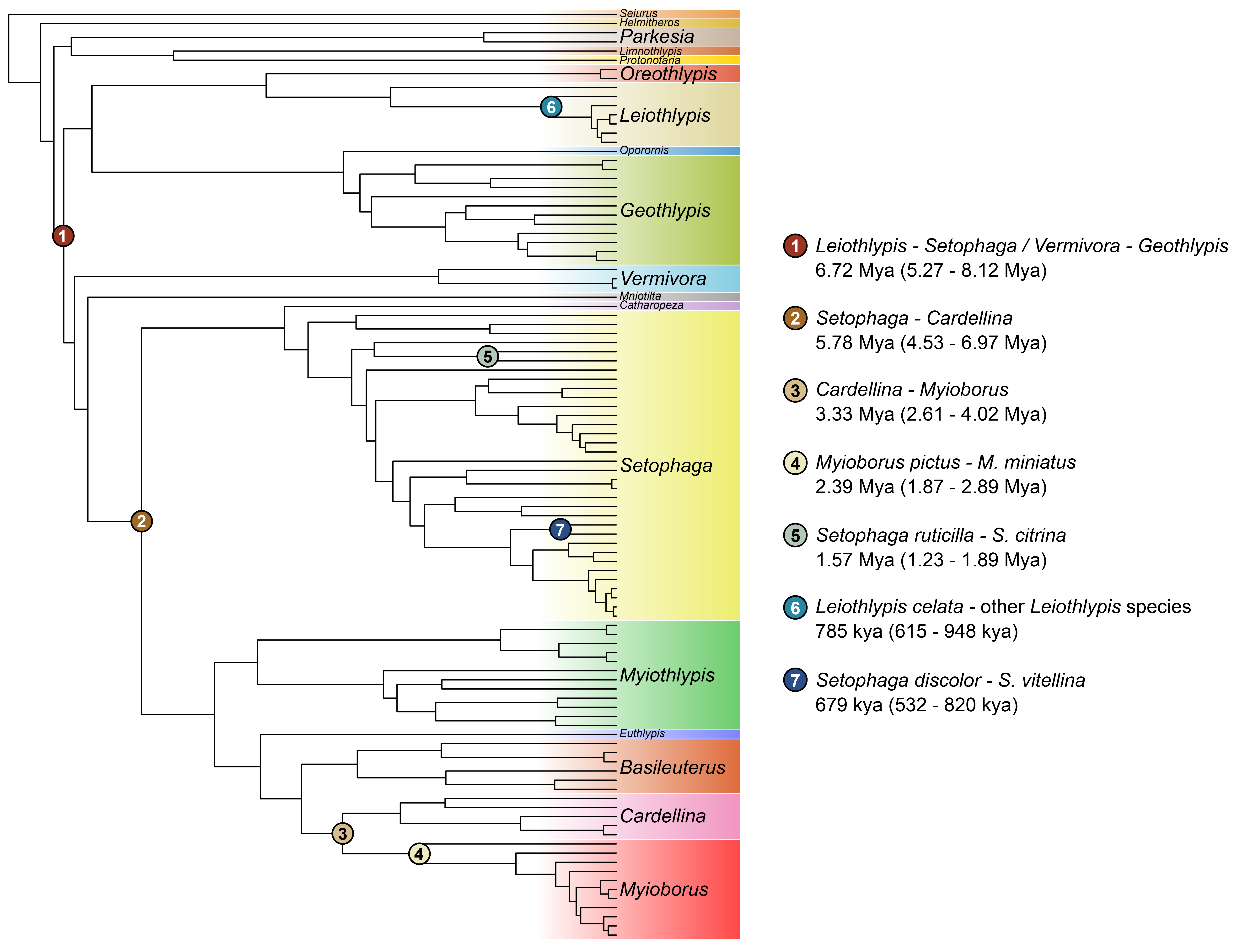

Supplement: S3 Fig — Intergeneric hybridization occurred between Leiothlypis and Setophaga (node 1), Setophaga and Cardellina (node 2), Vermivora and Geothlypis (node 1), and Cardellina and Myioborus (node 3). Introgression also occurred between Myioborus pictus and M. miniatus (node 4). BCO2 introgression from Leiothlypis to Setophaga likely occurred between nodes 5 and 7. Node 6 indicates that an ancestor of most extant Leiothlypis species may have been the gene flow donor. The data and code needed to generate this figure can be found at https://doi.org/10.5061/dryad.1zcrjdg3v and from NCBI at BioProject PRJNA630247. (PNG) [file pbio.3003501.s004.png]

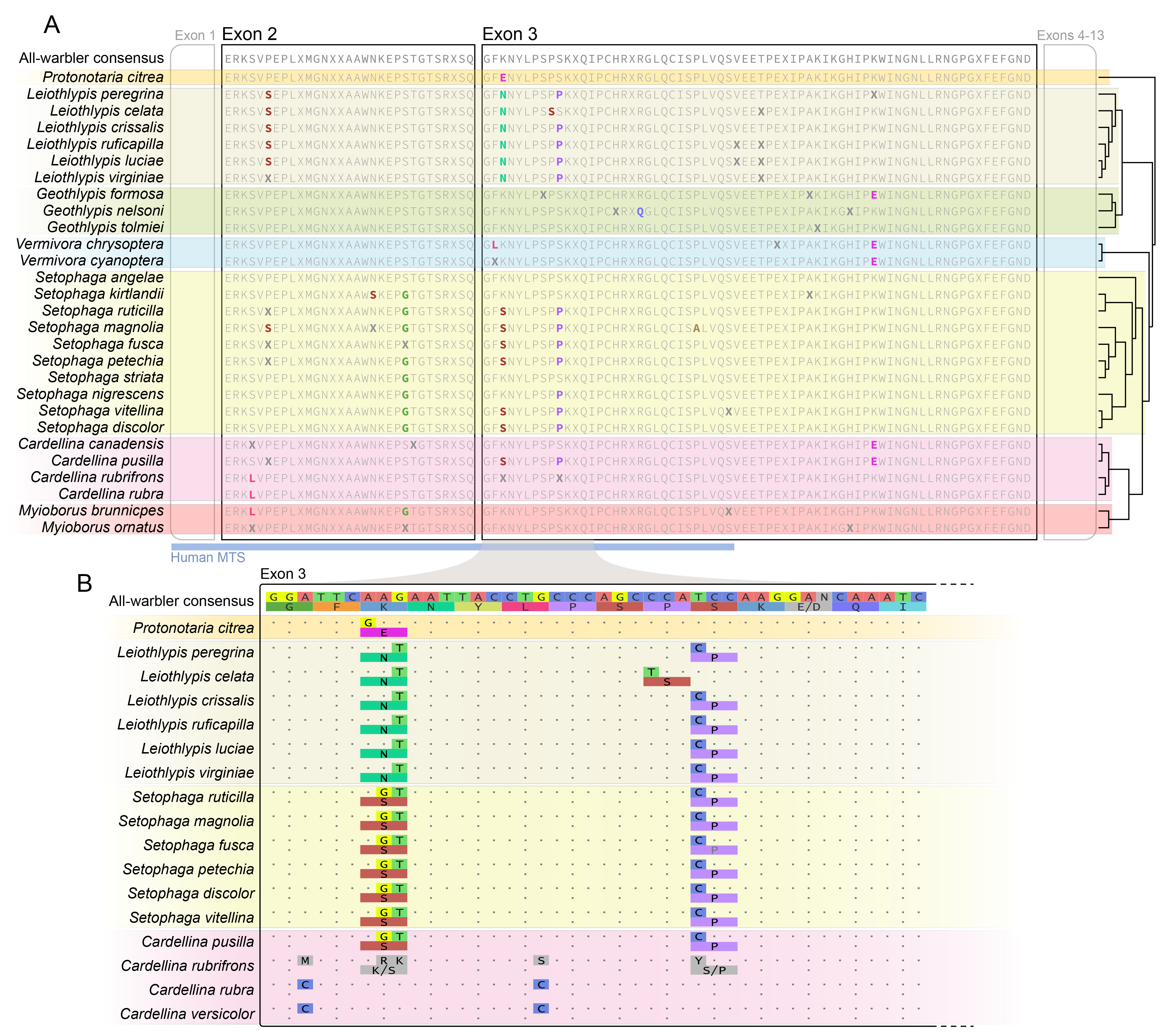

Supplement: S4 Fig — (A) BCO2 amino acid alignment of species involved in introgression and several relatives, showing the second and third exons. No amino acids are either unique to Vermivora and Geothlypis formosa or unique to Leiothlypis and the introgressed Setophaga and Cardellina species. Within-species variation is shown as an X. The sequence corresponding to the human mitochondrial targeting sequence (MTS) is indicated with a blue bar. (B) Alignment of the BCO2 coding sequence and its translation at the start of the third exon. The codon of interest is the third in the exon. Introgressed species likely inherited asparagine from Leiothlypis, which was subsequently replaced by serine after an A > G mutation in the second codon position. (PNG) [file pbio.3003501.s005.png]

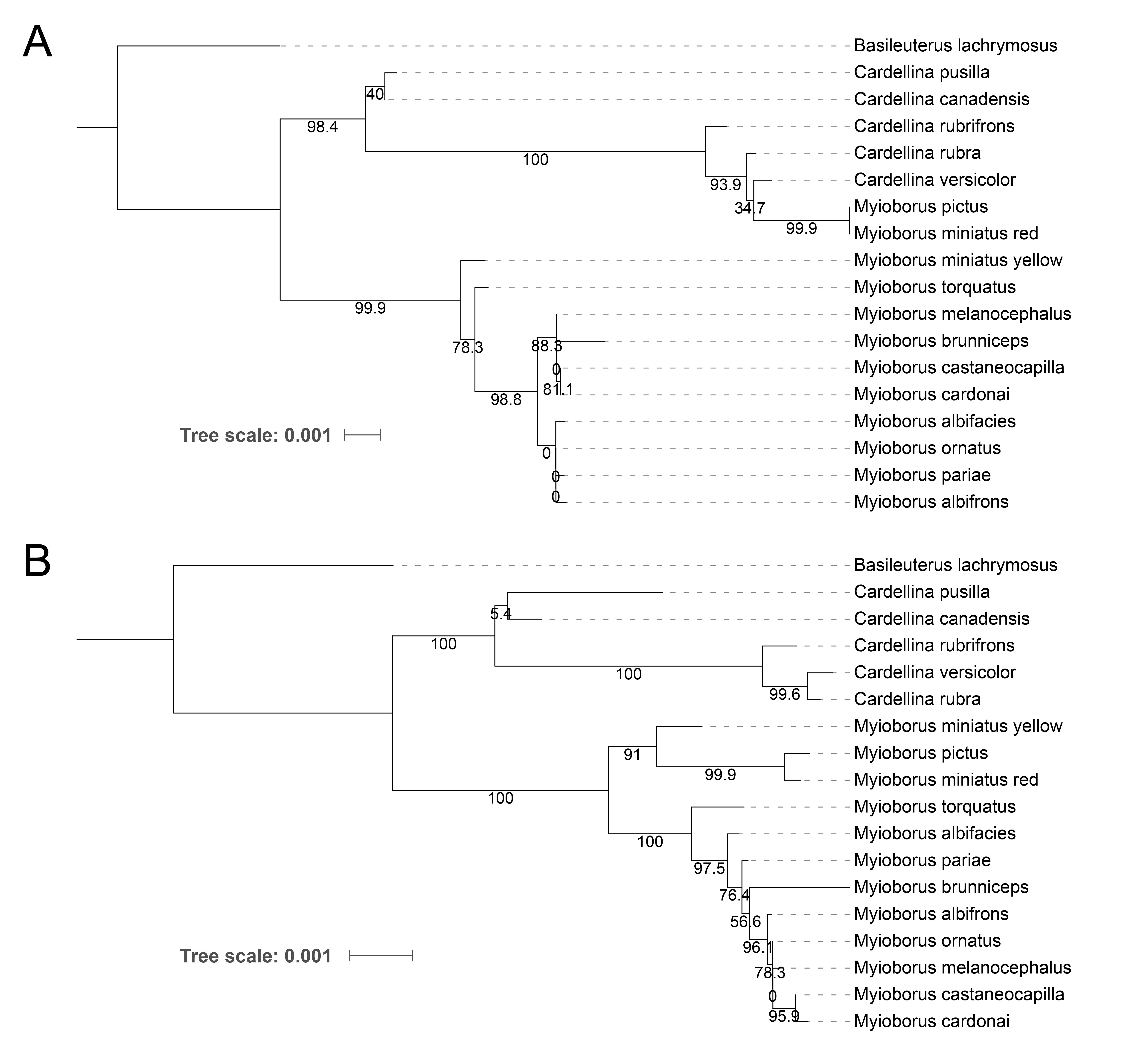

Supplement: S7 Fig — (A) Tree estimated from a 10.9 kb region of chr. 3 within and upstream of BDH1L. (B) Tree estimated from a 36.9 kb region of chr. 8 within and upstream of CYP2J19. Support values were calculated by 1,000 replicates of an approximate likelihood ratio test (aLRT). The data and code needed to generate this figure can be found at https://doi.org/10.5061/dryad.1zcrjdg3v and from NCBI at BioProject PRJNA630247. (PNG) [file pbio.3003501.s008.png]

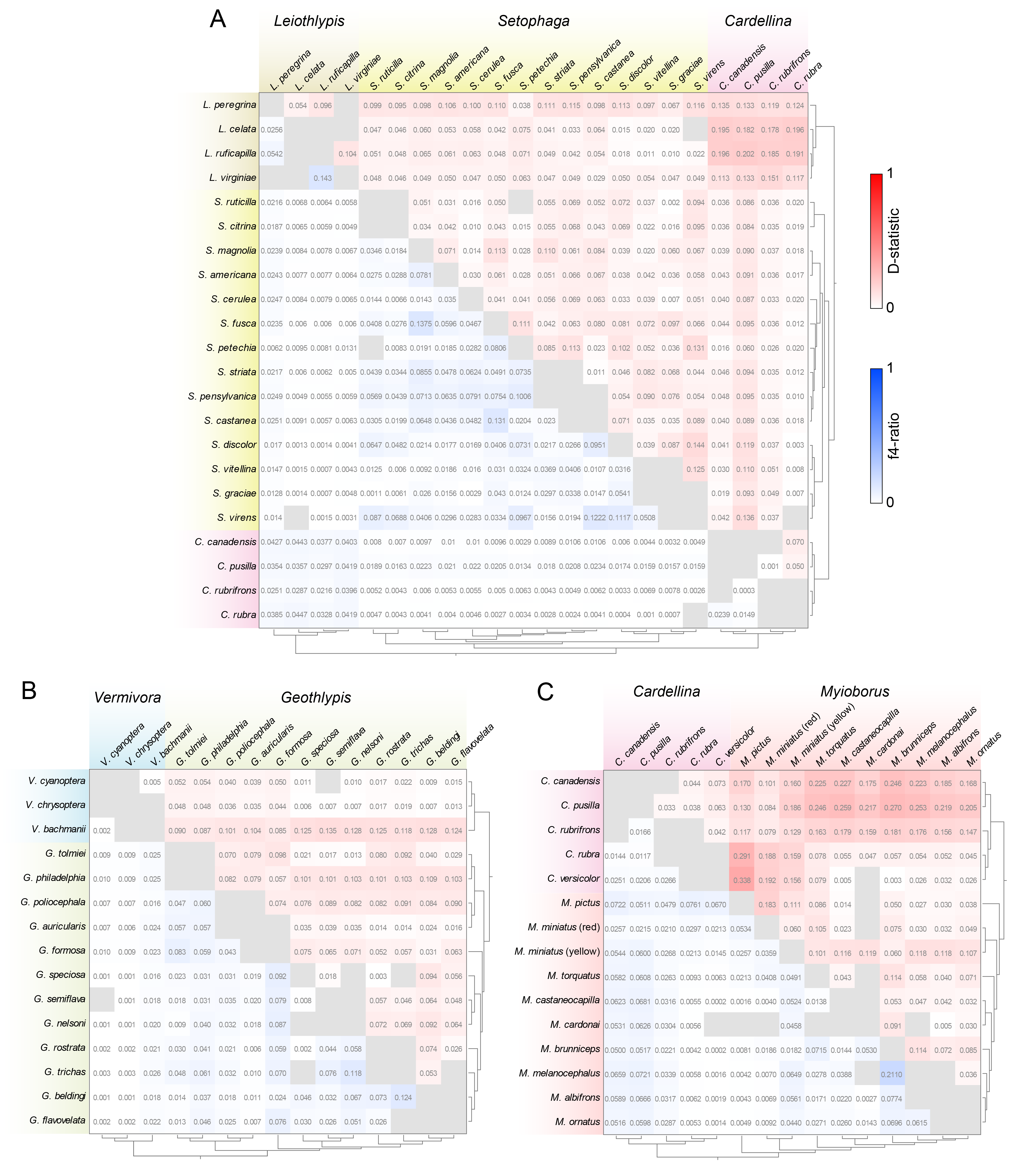

Supplement: S8 Fig — The D-statistic, above the diagonal in red, indicates a signal of shared alleles between two taxa. The f4-ratio, below the diagonal in blue, indicates the proportion of admixture in the genome between two taxa in the genome. (A) Leiothlypis, Setophaga, and Cardellina, including the species suspected to be involved in BCO2 introgression and several relatives. (B) Vermivora and Geothlypis. (C) Cardellina and Myioborus. The data and code needed to generate this figure can be found at https://doi.org/10.5061/dryad.1zcrjdg3v and from NCBI at BioProject PRJNA630247. (PNG) [file pbio.3003501.s009.png]

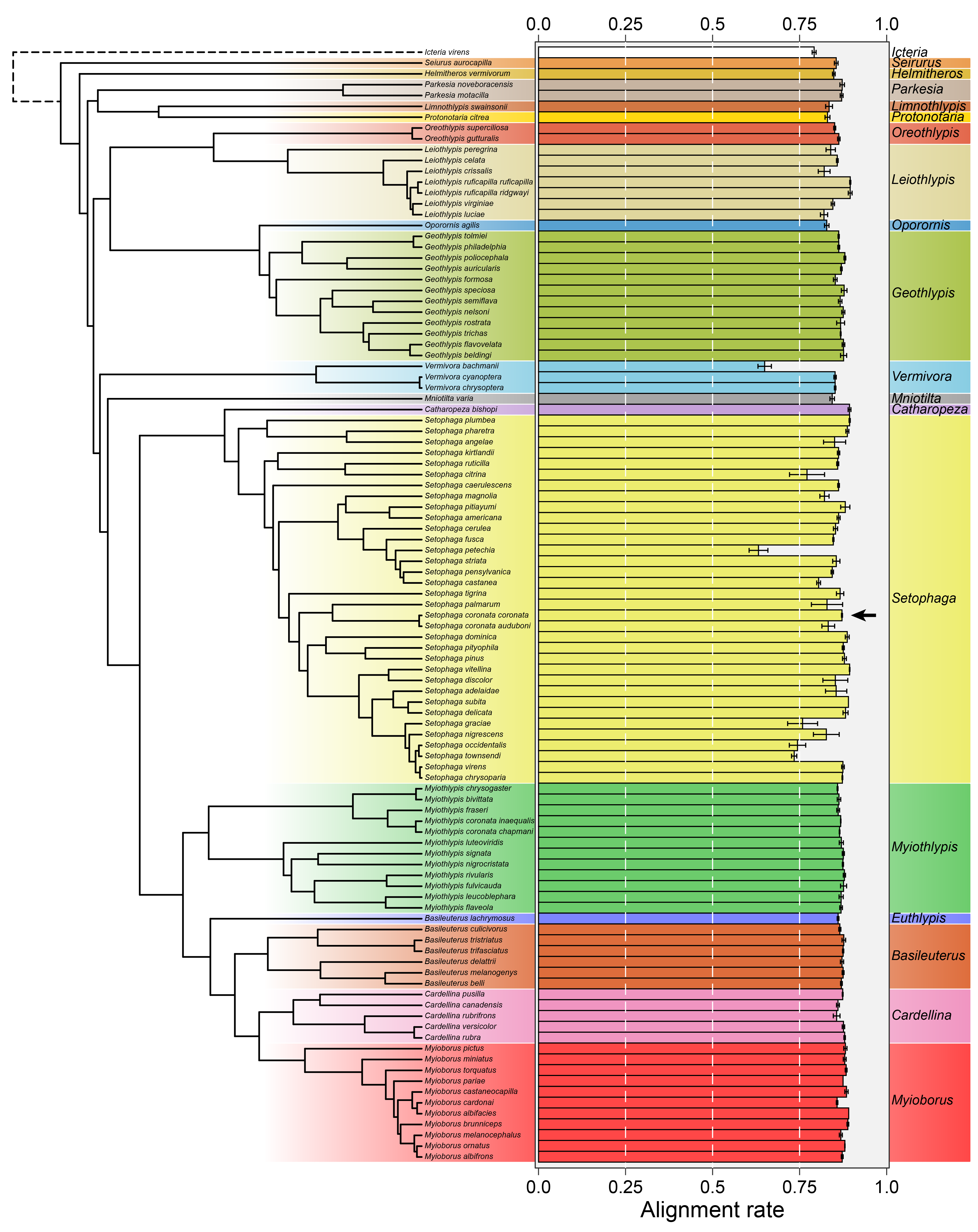

Supplement: S9 Fig — Error bars are standard errors. There is no reference bias or phylogenetic signal apparent in the alignment rates, which might be observed if species more diverged from the reference (S. coronata, denoted with a black arrow) showed lower alignment rates. The data and code needed to generate this figure can be found at https://doi.org/10.5061/dryad.1zcrjdg3v and from NCBI at BioProject PRJNA630247. (PNG) [file pbio.3003501.s010.png]

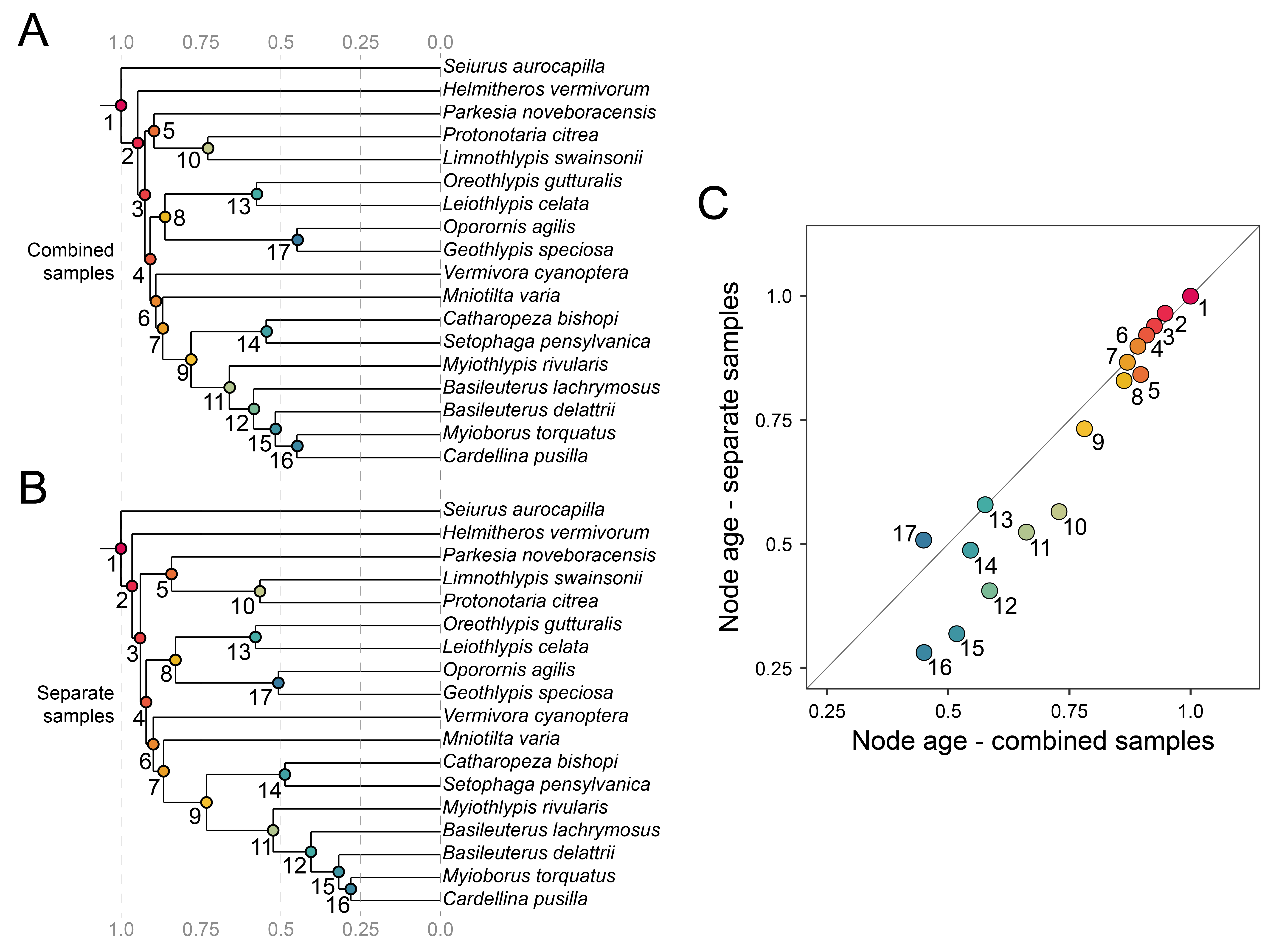

Supplement: S10 Fig — (A) The final concatenated ultra-conserved element (UCE) tree from main text Fig 1 subset to one species per genus. (B) A concatenated UCE tree made using three individuals per species from one species per genus. (C) Comparison of relative node ages between the two trees. Ages are fairly similar, but younger nodes are comparatively older in the combined-individuals tree. The data and code needed to generate this figure can be found at https://doi.org/10.5061/dryad.1zcrjdg3v and from NCBI at BioProject PRJNA630247. (PNG) [file pbio.3003501.s011.png]

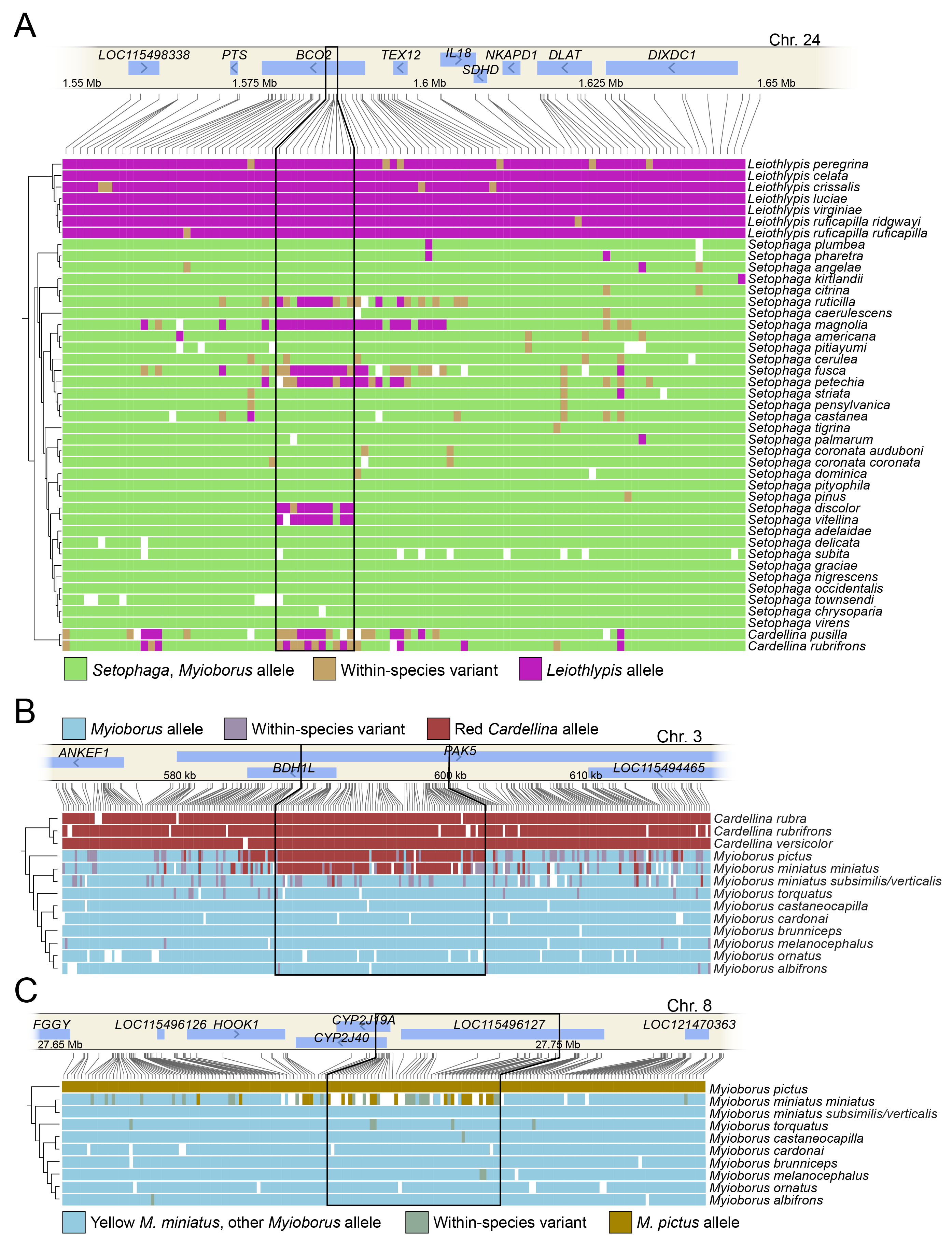

Supplement: S11 Fig — (A) BCO2, (B) BDH1L, (C) CYP2J19. The data and code needed to generate this figure can be found at https://doi.org/10.5061/dryad.1zcrjdg3v and from NCBI at BioProject PRJNA630247. (PNG) [file pbio.3003501.s012.png]

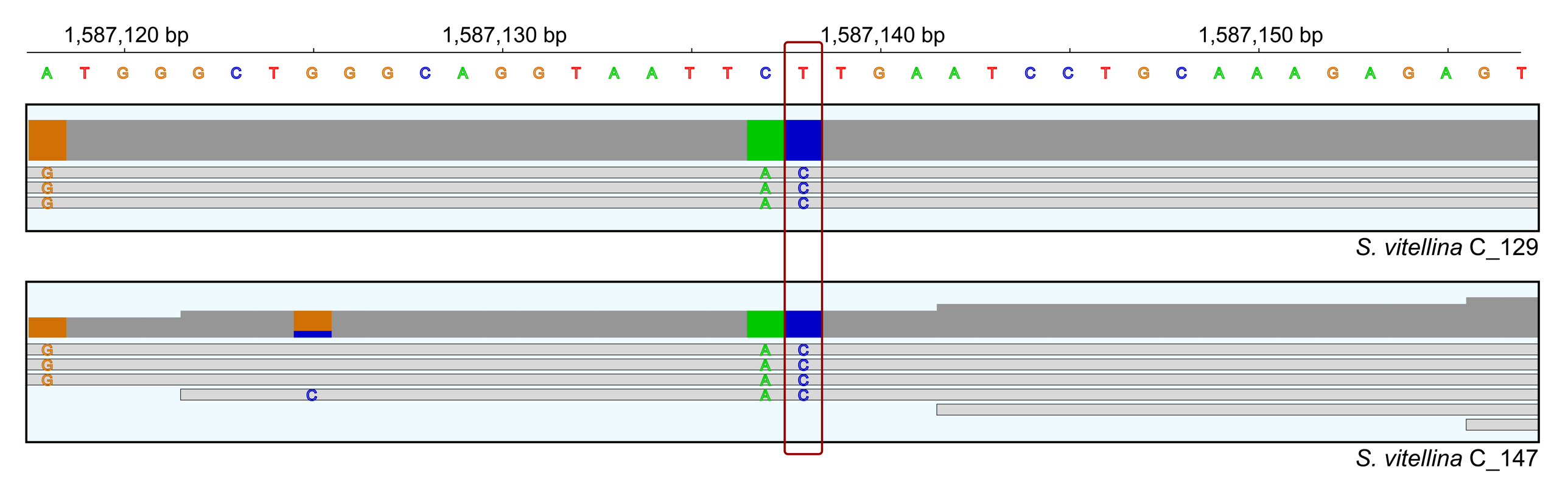

Supplement: S12 Fig — Although the genotype was uncalled in the master VCF file, every read at the position contained the allele from the BISC introgression haplotype. Because the gene is in reverse orientation in the S. coronata assembly, it is shown here as C, rather than G. Figure adapted from IGV [81]. (PNG) [file pbio.3003501.s013.png]
